# Thoracoscopic surgical management of esophageal benign tumor

**DOI:** 10.1186/1749-8090-8-S1-O258

**Published:** 2013-09-11

**Authors:** HW Jeon, SW Sung, JK Park, H Song

**Affiliations:** 1 Thoracic and Cardiovascular Department, Bucheon St. Mary's Hospital, Bucheon, Republic of Korea

## Background

Benign esophageal tumors are rare; complete surgical resection is essential for the management of the submucosal tumors. Larger, symptomatic, or non-diagnostic lesions should be resected for both diagnostic and therapeutic indications. VATS has become a popular treatment in the thoracic surgery, however thoracoscopic esophageal surgery may lead to increased operative complications. We evaluated the effect and safety of thoracoscopic surgery foe esophageal submucosal lesions..

## Methods

A retrospective study evaluated patients undergoing thoracoscopic treatment of benign submucosal tumors. Between March 2011 and Feburary 2013, 16 patients underwent thoracoscopic resection of benign submucocal tumors. Intraoperative esophagoscopy was performed for tumor localization by transillumination and confirmation of mucosal integrity after enucleation in every patient.

## Results

Median patient age was 46.5 years (range 30-56). The median surgery time was 167.5 minutes (range: 80-429). The median tumor size was 3.65 cm (range 1.3-9). The median hospital stay was 4 days (range 2-12). There were 15 leiomyoma 1 neurogenic tumor. There was one case of conversion to thoracotomy due to residual tumor after enucleation by esophagoscopy. Mucosal injuries occurred in three patients, two accindentally and one intentionally; each patient was treated with primary repair and confirmed integrity with flexible gastroscopy. We could localize the small tumor in four patients with esophagoscopy. Esophagoscopic assistance was necessary in eight patients to have better idea where to make myotomy. There were no major morbidities such as postoperative leakage or mortality.

## Conclusion

Esophagela submucosal tumors can be treated safely with thoracoscopic surgery. However intraoperative esophagoscopy allows more accurate tumor localization and direction of esophageal access incision and decreases complications during VATS enucleation of esophageal submucosal tumors.

